# Family case of Potocki-Lupski syndrome

**DOI:** 10.1186/s13039-024-00673-5

**Published:** 2024-03-22

**Authors:** L. N. Kolbasin, T. A. Dubrovskaya, G. B. Salnikova, E. N. Solovieva, M. Yu. Donnikov, R. A. Illarionov, A. S. Glotov, L. V. Kovalenko, L. D. Belotserkovtseva

**Affiliations:** 1 Budgetary Institution of KHMAO-Yugra Surgut Regional Clinical Center for Maternity and Childhood Protection, Medical Genetic Counseling Service, Surgut, Russian Federation; 2 Budgetary Institution of KHMAO-Yugra “Kondinsky Regional Hospital”, Mezhdurechensky Town, Russian Federation; 3 Budgetary Institution of Highest Education of KHMAO-Yugra “Surgut State University”, Surgut, Russian Federation; 4 D.O. Ott Research Institute of Obstetrics, Gynaecology, and Reproductology, 199034 St. Petersburg, Russian Federation

**Keywords:** Potocki-Lupski syndrome (PTLS), Family case study, 17p11.2 microduplication

## Abstract

**Background:**

Potocki-Lupski syndrome (PTLS, OMIM # 610883) is a rare genetic developmental disorder resulting from a partial heterozygous microduplication at chromosome 17p11.2. The condition is characterized by a wide variability of clinical expression, which can make its clinical and molecular diagnosis challenging.

**Case presentation:**

We report here a family (mother and her two children) diagnosed with PTLS. When examining children, neurological and psychological (neuropsychiatric) manifestations (speech delay, mild mental retardation), motor disorders, craniofacial dysmorphism (microcephaly, dolichocephaly, triangular face, wide bulging forehead, long chin, antimongoloid slant, "elfin" ears) were revealed. The suspected clinical diagnosis was confirmed by MLPA and CMA molecular genetic testing which revealed the presence of a segmental aneusomy; microduplication in the 17p11.2 region.

**Conclusions:**

Children with PTLS can have a clinically recognizable and specific phenotype: craniofacial dysmorphism, motor and neurological manifestations, which may implicate a possible genetic disease to the attending physician. Moreover, each child with this syndrome is unique and may have a different clinical picture. The management of such patients requires a multidisciplinary team approach, including medical genetic counseling.

## Introduction

In medical practice the term "orphan disease" for many doctors is equivalent to the meaning "rare condition". This is due to the fact that orphan diseases in the work of doctors are, in their opinion, rare. Indeed, the prevalence of these diseases is not high and is less than 10 cases per 100,000 population of Russia. However, the exact number of orphan diseases is unknown. Presumably, there are more than 7000 of them. The Ministry of Health of the Russian Federation includes 267 groups of diseases in the list of orphan diseases presented on the Internet portal of the department [1].

Numerical chromosomal rearrangements, such as, for example, aneuploidy syndrome trisomy 21, are not orphan diseases and are not uncommon in the population, and their specific clinical diagnosis usually requires a standard cytogenetic laboratory study. The detection of structural anomalies of chromosomes depends on their characteristics (size, localization, type of rearrangement), as well as the method used to interrogate the human genome for the clinical laboratory study. In this case, the resolution of conventional G-banded karyotyping cytogenetic study is around 5–7 million base pairs. With the introduction of modern molecular cytogenetic technologies, such as comparative genomic hybridization (chromosomal microarray, CMA), chromosome studies have become possible with a resolution of 1,000 base pairs or less. This has made it possible to detect submicroscopic rearrangements and refine the coordinates of genomic disorders, including microdeletions and microduplications [2]. The widespread use of CMA and multiplex ligase-dependent amplification (MLPA) for copy number changes of small genomic segments has expanded the understanding of the causes of some syndromes and disorders.

One of these conditions is the relatively recently described syndrome of partial microduplication of a segment of the short arm of chromosome 17 (17p11.2). This duplication, most commonly 3.7 million base pairs in size, results from non-allelic homologous recombination at sites rich in low-copy repeats ranging in size from 10 to 400 thousand base pairs with 95–97% sequence identity. This region contains more than 20 disease-associated genes, the key contributing gene for the development of the disease is *RAI1*. A duplication of the above size occurs in 66% of cases, in other cases the duplicated region has a length of 0.41 to 19.7 million base pairs, but always includes the *RAI1* gene [3–5]. This microstructural rearrangement was first described in 1996. In 2000, the first clinical study of this disease was published, and by 2007 enough patients had been collected to complete a comprehensive study and a detailed description of the syndrome. The names of the two researchers who presented it, Lorraine Potocki and James R. Lupski from Baylor College of Medicine (USA), were included in the eponymous name of the syndrome (PTLS) [6–8].

Despite the fact that PTLS is not included in the list of orphan diseases approved by the Russian Ministry of Health, it is considered as a rare condition with the incidence not exceeding one case per 25,000 people [3]. At the same time, about 1,000 patients with PTLS have been diagnosed worldwide to date [9]. In the available Russian scientific literature there are single descriptions of cases of this syndrome [10]. Most observations of families with this syndrome are found in foreign sources. In the United States, there is a PTLS Families Support Fund that collects information about patients and has published a brochure for them [9]. There are, however, two reported cases of transmission of the PTLS associated with 17p11.2 duplication. Nevertheless, in Russia there is no separate register of families with PTLS, so the frequency of the disease and the number of registered cases is not known.

The clinical phenotype of PTLS patients is characterized by mild dysmorphic facial features, hypermetropia, infantile hypotension, delayed psychoverbal and physical development, mental retardation of varying severity, autism spectrum disorders, behavioral anomalies, sleep apnea, and cardiovascular anomalies [11]. The syndrome is described by most researchers as clinically heterogeneous, with variability of expression with a wide range of clinical severity and the absence of obligatory pathognomonic signs, which makes its clinical diagnosis challenging. Moreover, the vast majority of publications are devoted to children, so the phenotypic spectrum in adults with PTLS is not well defined. PTLS in most cases occurs sporadically, but can be inherited in an autosomal dominant manner, indicating a 50% risk of recurrence of the disease in the family [12].

In our clinical cytogenetic opinion, in order to increase knowledge and training of related experts and to improve the diagnosis and monitoring of rare genetic diseases, it is important to document such cases. This is especially important for practicing clinical doctors of regional and remote healthcare providers. Below we describe interesting clinical case study of familial PTLS, registered in a single Yugra family with two children and their mother.

## Materials and methods

Genomic DNA was extracted using column-based “Blood DNA Mini Kit” (Foregene, PRC). MLPA was performed according to the manufacturer’s instructions using SALSA Probemix P245-B1 Microdeletion Syndromes-1A (MRC-Holland, the Netherlands). Fragment analysis was performed on Nanophor 05 genetic analyzer (Syntol LLC, Russia). MLPA raw data analysis was performed using GeneMarker software v3.0.0 (SoftGenetics LLC, USA). CMA was performed using GenetiSure Pre-Screen Microarray Kit G5963A (8 × 60 K) (Agilent, USA) according to manufacturer’s instruction. Data analysis was performed using software Agilent Cytogenomics v5.1.2.1.

## Case presentation

The patient has been ascertained and monitored by a Surgut Regional Clinical Center for Maternity and Childhood Protection (Surgut, Russia). The study was approved by the Institutional Review Board of Surgut State University of the KHMAO—Yugra (Surgut, Russia), No. 17 from 2021–10-21. Written informed consent for the research was obtained from all study subjects. The study was performed in accordance with the Declaration of Helsinki.

### Child F. (boy), born in 2016

A child from the first pregnancy (mother's age was 23 years old), which occurred against the background of myopia and weight loss, chronic placental insufficiency and fetal growth retardation syndrome, the first premature, rapid birth, in breech presentation, at a gestational age of 33 weeks. The child's Apgar score was 4/8 points. The child's body weight at birth was 1570 g (0.1‰); length 41 cm (0.1‰); chest circumference 26 cm; head circumference 32 cm (0.26‰). Immediately after birth, resuscitation was carried out due to respiratory disorders.

During the first 11 days of life the child was observed in the ICU on an artificial lung ventilation and after that was further observed in the neonatal pathology unit. On the 41st day of life the child's condition stabilized, body weight reached 2,090 g, length 43 cm. He was discharged with a diagnosis of: severe ischemic – hemorrhagic damage of the central nervous system, periventricular and intraventricular hemorrhages of the 2nd degree, syndrome of movement disorders, choroid plexus cyst of the right lateral ventricle, severe respiratory distress syndrome, anemia of prematurity. Mental and motor development delay was noted during control examinations at all scheduled dates, *e.g.* he began to hold his head by 6 months, at 12 months he did not yet sit on his own, did not roll over, did not crawl; his speech was represented by vowels in the form of cries, no babble was registered. At the age of one year he was recognized as a disabled child with a diagnosis of astatic-atonic form of cerebral palsy.

At the age of 20 months despite the previously established clinical diagnosis he began to sit up on his own and crawl in a crouching manner. At two years old he was restless in behavior and easily distracted. He did not respond to verbal instructions, did not know how to play with toys, threw all the objects, hit them on the furniture. He couldn't eat with a spoon. The gait was ataxic: he spread his legs wide, pushed his pelvis forward to maintain balance, periodically moved on all four extremities. He partially understood the spoken speech. His own speech was represented by cries, mostly vowels. There was no imitative speech. He did not show animals in the picture. He ripped pages from a book. Hypersalivation was noted, saliva was not swallowed. Muscle tone was diffusely reduced in the extremities. He began to walk independently at the age of 2 years 8 months, at the same time he began to pronounce the words “mother”, “dad”. Often for a long time he made stereotypical monotonous movements (swinging his body, nodding his head), crushing and twisting his lips and tongue with his hand. Sometimes at the same time he inflicted self-harm (scratches, abrasions). According to his mother, he was aggressive towards his own younger sister and tried to hit her.

**Fig. 1:**
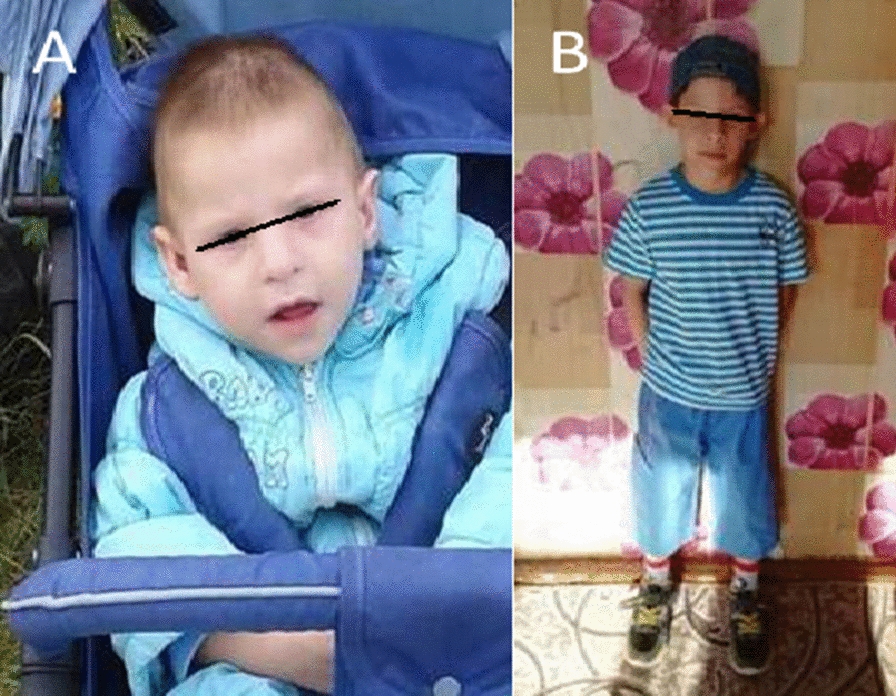
Child F., aged 1 year 5 months (**A**), 6 years old (**B**) (elongated skull, microcephaly, short palpebral fissures, antimongoloid slant, convex forehead, long chin, triangular face, wide bridge of the nose, “elfin” ears)

Minor developmental anomalies attracted the attention of the pediatrician: an elongated skull, microcephaly, short palpebral fissures, antimongoloid slant, convex forehead, long chin, triangular face, wide bridge of the nose, “elfin” ears (Fig. 1). At six years old the child's height was 113 cm, body weight—16.3 kg, head circumference—46 cm, body mass index (BMI)—12.7 kg/m^2^ (SDS BMI—1.13 SD). The child had a deficiency of body weight, microcephaly (OFC z-score < -2).

### Child T. (girl), born in 2020

The child was from the third pregnancy which proceeded against the background of anemia in the first and second half, feto-placental insufficiency, false contractions before and after 37 weeks, the third urgent, physiological birth at 39 weeks 5 days of gestation. Diagnosis at birth: retardation of intrauterine development. Double tight entanglement of the umbilical cord around the neck. The child's Apgar score was 3/8 points. She screamed after tactile stimulation. Body weight at birth was 2418 g (2.3‰); length 50 cm (67.7‰); head circumference 32 cm (0.26‰); chest circumference 31 cm. Neonatal jaundice developed on the third day, and toxic erythema developed on days 3–4. Diagnosis at discharge from the hospital: intrauterine growth retardation, hypotrophic type. She was breastfed for up to 1 month.

At the age of 15 days she was admitted to the pediatric department with acute unspecified nasopharyngitis, neonatal conjunctivitis and challenged feeding. She was taken to the hospital by air ambulance in a state of moderate severity and was on treatment for 10 days. After discharge observation at the place of residence in the local hospital was continued. She was moved to mixed feeding (breast fed plus bottle fed).

At the age of 7 and 10 months she twice suffered from coronavirus infection and was twice admitted in the infectious diseases department. During the second admission the girl was examined by a neurologist which determined signs of physical development delay. According to the mother developmental delay was noted initially. She began to hold her head from the age of 4 months and until the time of the examination she did not sit on her own, did not crawl, did not stand at the support, did not walk with support by the hands.

**Fig. 2:**
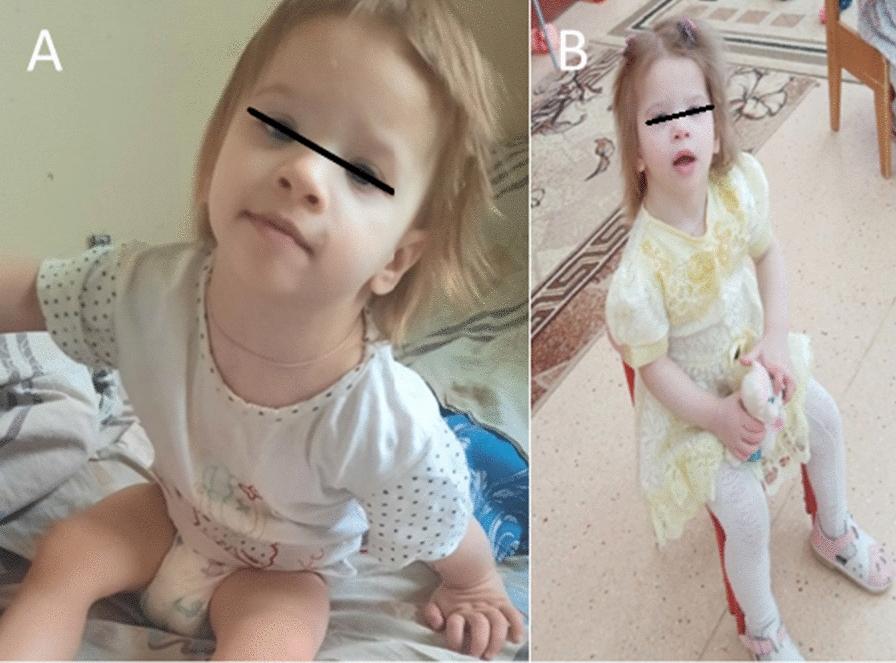
Child T., born in 2020, aged 1 year 7 months (**A**), 2 years 5 months (**B**) (elongated skull, short palpebral fissures, antimongoloid slant, convex forehead, long chin, triangular face, broad bridge of the nose)

At the age of 1 year 7 months the child was examined in a hospital for the purpose of primary medical and social assessment using laboratory tests and imaging. The child was diagnosed with motor and behavioral disorders, delayed physical and mental development: motor disinhibition (constant moving around the ward without a specific goal). Everything she grabbed was thrown to the floor or knocked on the surface. She used toys for other purposes, *e.g.* she gnawed a polyurethane ball, bit off pieces, did not enter the game, did not understand how to play with the ball (toss, throw, roll); she knocked with a plastic doll on the nightstand. The gait was ataxic: the legs were widely spaced, the pelvis was brought forward, the body was tilted back. She couldn't get on the bench. She did not walk up the stairs. She did not use cutlery, she couldn’t drink from a mug. She held the bottle with two hands. Requests were not fulfilled. She did not respond to either comments or verbal limitations. She couldn't chew. She ate mashed or crushed food. Speech was represented by vowels in the form of cries. The muscle tone of the extremities was diffusely reduced. There is a fist grip in the handles, thumb opposing is formed. There was no pinch grip. She did not ask for a potty, she did not understand the purpose of the pot, physiological excretions are made in diaper. According to the Griffith psychomotor development scale a total score of 167 points was scored, which corresponded to the age of 13 months (Fig. 2). Analyzing the data obtained during the next study of a child at the age of 19 months it was possible to note her lag in psychomotor development for all functions by the age of 5–6 months. As with the affected sibling, minor developmental anomalies were noted by the clinical observer: an elongated skull, short palpebral fissures, an antimongoloid slant, a convex forehead, a long chin, a triangular face, and a wide bridge of the nose. At the age of 2 years 5 months the child's height was 89 cm, body weight—11.8 kg, head circumference—45 cm, BMI—14.9 kg/m2 (SDS BMI − 0.7 SD). The child has microcephaly (OFC z-score < − 2).

### Family history

**Fig. 3:**
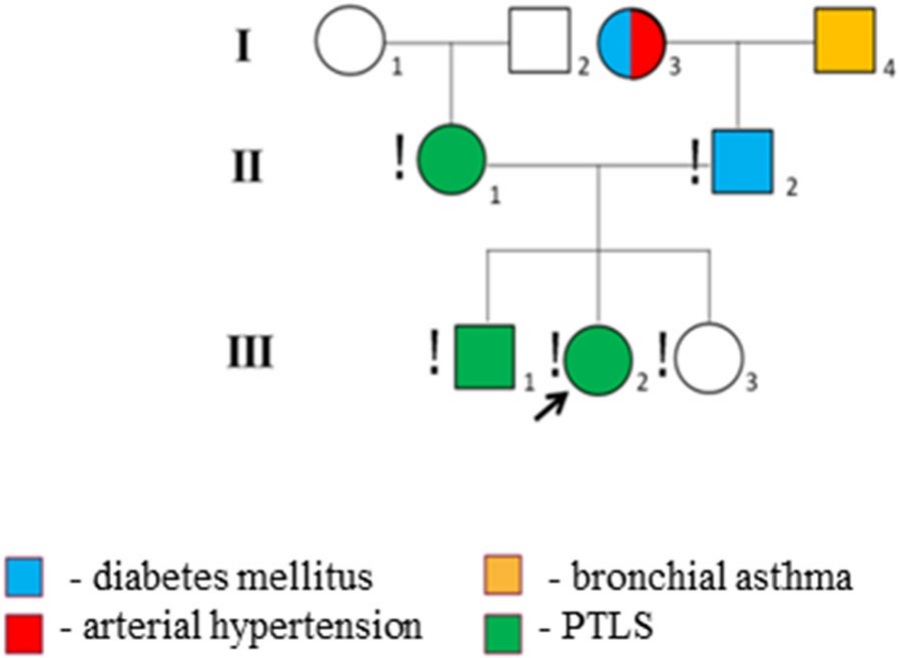
Family pedigree. **!** Means that the person was examined by geneticist

The mother of the children, a 28 years old, was diagnosed clinically with: myopia, 1st degree scoliosis and chronic iron deficiency anemia, whereas the father of a child (31 years old) suffers from type 2 diabetes mellitus. According to the parents, the paternal grandmother suffers from type 1 diabetes mellitus, arterial hypertension, and the paternal grandfather has bronchial asthma (Fig. 3). The family history is hereditary tainted (D/N ratio = 0.75, where D is the total number of diseases for all known proband’s relatives, N—total number of proband's relatives).

**Fig. 4:**
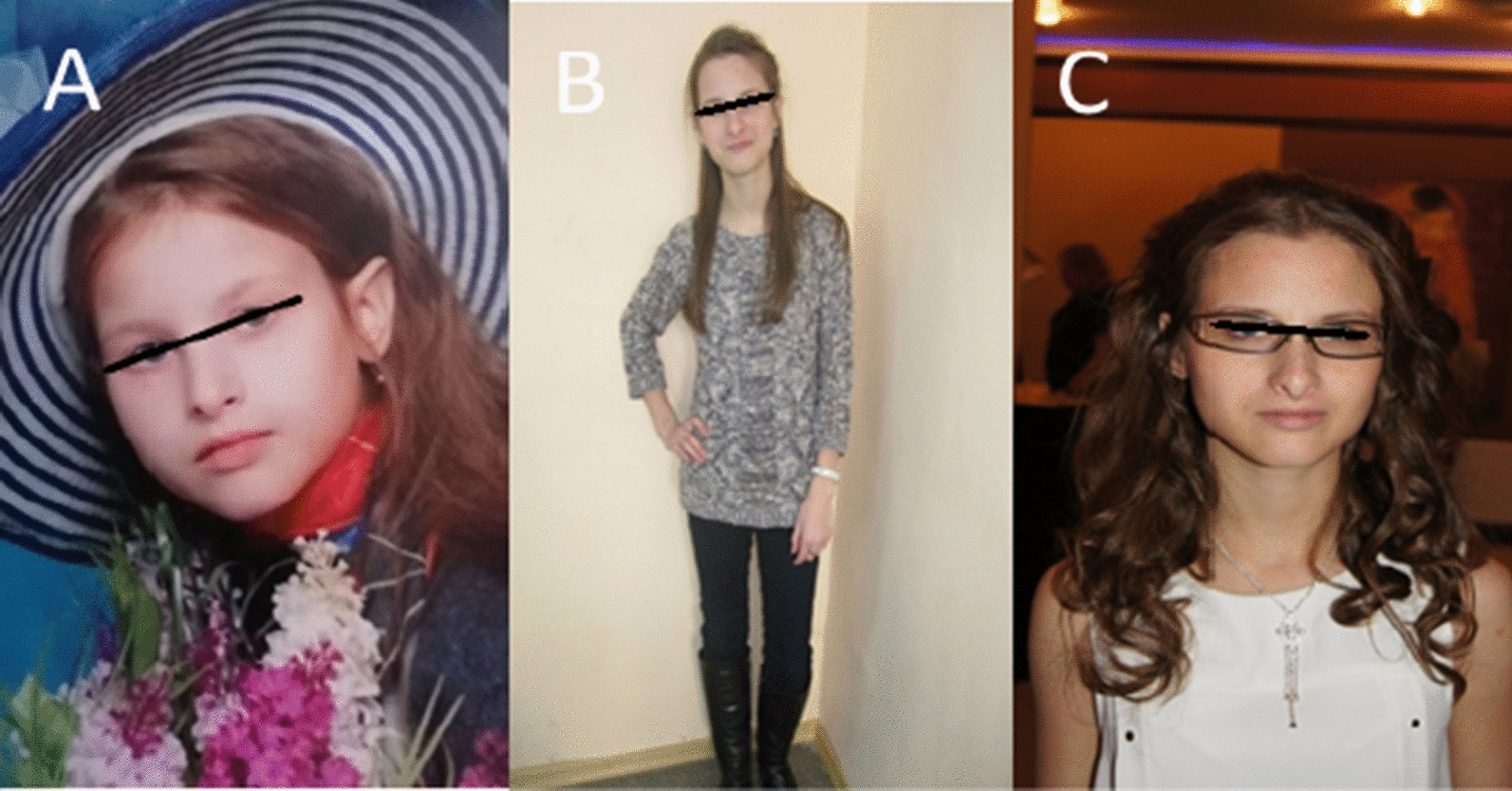
Mother II-1 at the age of 10 (**A**), 17 (**B**) and 21 (**C**) years old (elongated skull, prominent forehead, palpebral fissures slightly tilted downward, “elfin” ears)

According to II-1, she had a neuropsychiatric developmental delay in her childhood, and she was registered with a neurologist at the dispensary. Despite the delay in development, she graduated from the 9th grade of a secondary school with in-depth learning of English, an industrial and humanitarian college and received the profession of a catering technologist (Fig. 4).

### Clinical laboratory investigation

General clinical laboratory examination of children (general blood tests, urine, biochemical parameters of liver and kidney function), ultrasound examination of the abdominal cavity and retroperitoneal space, cardiological examination (including electrocardiography and echocardiography) did not reveal any clinically significant pathological changes.

**Fig. 5:**
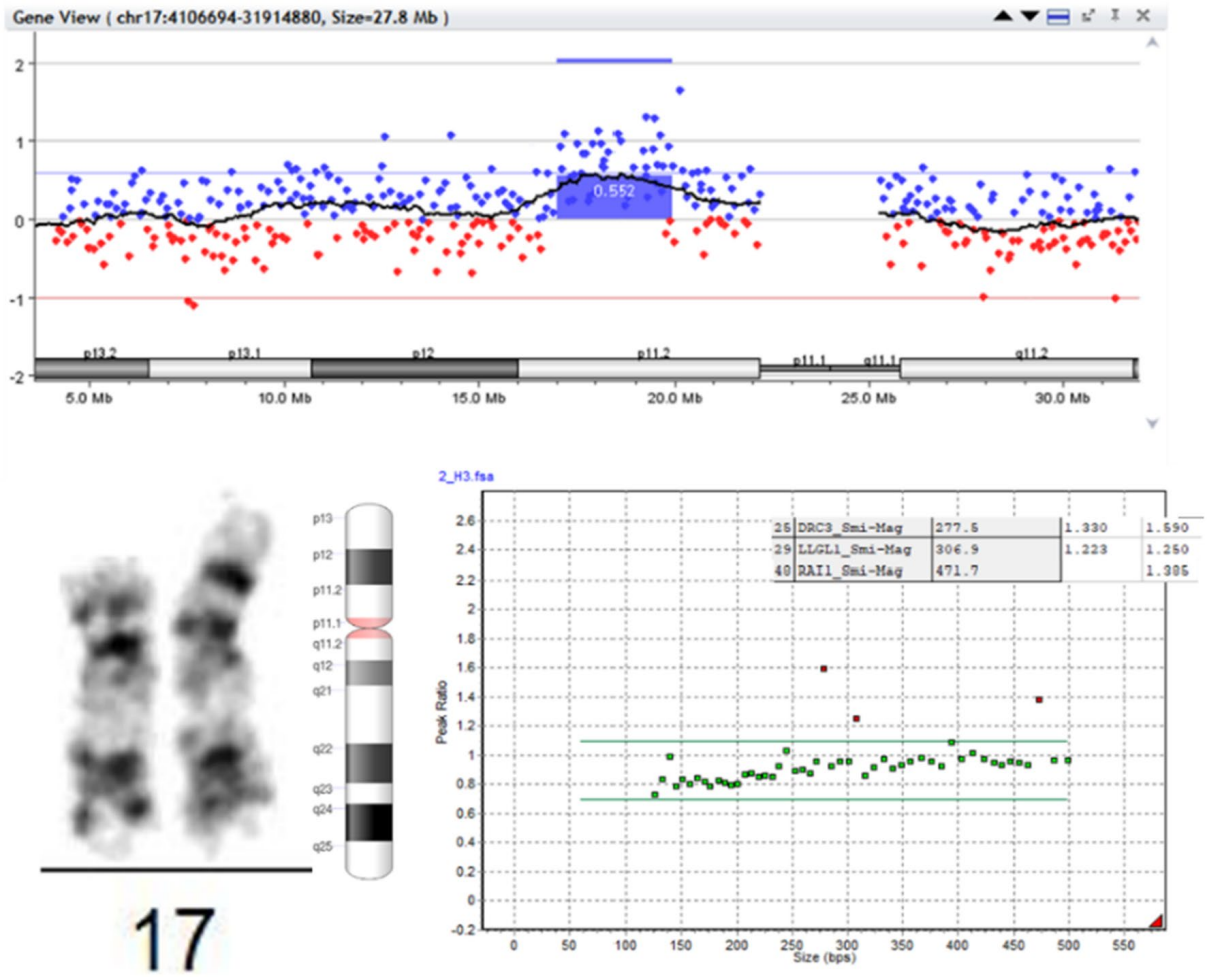
Results of cytogenetic and molecular studies. **a** CMA plot showing the 2,9-Mb duplication of 17p11.2 region: arr[GRCh37] 17p11.2(17019267_19947934) × 3 found in mother, daughter and son. **b** Typical partial G-band karyotype obtained from peripheral lymphocytes of PTLS family with 450 GTG bands showing dup(17) (p11.2). **f** MLPA analysis of the sequences involved in a distinct subset of microdeletion and microduplication disorders (SALSA® MLPA® Probemix P245-B1 Microdeletion Syndromes-1A; MRC-Holland, Amsterdam, The Netherlands), where our patients show duplication of *DRC3, LLGL1, RAI1* genes involved in PTLS formation. The same results were obtained for both studied sibs and their mother

Considering the hereditary history, the presence of a delay in physical and psychoverbal development and phenotypic features (small developmental anomalies), medical genetic counseling of the entire family was carried out in a regional medical genetic counseling service. All family members had a standard cytogenetic study that revealed changes observed in the karyotype, as well as a MLPA for *RAI1* performed for samples of genomic DNA. CMA also confirmed MLPA and karyotyping findings (Fig. 5). As a final result 17p11.2 microduplication was detected in children III-1 and III-2, which was also found in their mother II-1, and this fact indicated an autosomal dominant transmission of the identified rearrangement.

### Preliminary diagnosis

Based on clinical report from family, anamnesis data, objective examination of children and mother, medical genetic counseling, molecular genetic analysis, the children were diagnosed with Potocki-Lupski Syndrome (OMIM: 610,883), microduplication 17p11.2, autosomal dominant type of inheritance.

### Natural history

Specific optimized educational and therapeutic interventions for children with PTLS continues to evolve. Each child receives symptomatic individual treatment adapted to his or her needs, including physiotherapy, speech therapy, psychological and psychotherapeutic assistance. Collaboration between teams of clinical specialists in pediatrics, medical genetics, neurology, psychiatry, psychology and other clinical disciplines is essential when caring for these children.

Child III-1 at the age of 1 year 8 months was sent to a rehabilitation center for the purpose of rehabilitation treatment, which was difficult due to behavioral disorders. From the age of three, he attends a preschool institution at the place of residence. He did not play with other children, he wasn’t assiduous in class, he did not respond to verbal instructions. Over time, the dynamics of the child's gait became more established. He eats on his own with a spoon, drinks from a mug. Vocabulary at the age of 4 was 8–10 words. Child III-2 also successfully attends kindergarten.

### Prognosis

As a result of a multidisciplinary team approach (pediatrician, neurologist, speech therapist, psychiatrist, social care workers, and others), the likelihood of a favorable adaptation of children remains—acquiring self-care skills and the possibility of further socialization.

## Discussion

Several scientific articles devoted to the study of PTLS syndrome are similar to our described family case. Among these Grama et al. [3] reports the first Romanian family (a mother and her five children) diagnosed with PTLS (17p11.2 microduplication). A girl patient from Sri Lanka who was diagnosed with PTLS and Xq29 duplication at the age of 4 years was described by Sumathipala et al. [13]. According to review of Pratico et al. [14], about 50 cases of this syndrome have been published worldwide. We considered it necessary to present our case of a family PTLS disease, which has both similar and different descriptive features when comparing with cases already described in the scientific literature. Thus, we conducted a comparative analysis of the clinical and genetic data (Table 1) of our family case and three family cases described and published previously [3, 13, 14].

**Table 1:** .

Clinical and genetics data	Child 1	Child 2	Child 3	Child 4	Child 5	Mother	Child 1	Child 1	Child 1	Child 2	Mother
	Romanian family [3]						Sri-Lanka [13]	Italy [14]	Russia (Yugra region)		
Age at diagnosis, years	13	8.2	5.5	2.10	3.0	33	4	2.5	5	1.7	28
Sex	F	F	F	M	M	F	F	M	M	F	F
Insufficient feeding, BMI z-score, SD	No 0.2	Yes – 2.4	Yes – 2.8	Yes – 3.7	No 0.12	No 0.5	No	No	Yes 1.1	No 0.7	No
*Face features*											
Micrognathia	+	+	+	+	+	+	–	–	–	–	–
Downslanting palpebral fissures	–	–	+	+	+	–	+	+	+	+	+
Broad forehead	–	+	+	+	+	–	+	+	+	+	+
Pointed nose tip	+	+	+	+	+	+	+	–	+	+	+
Triangular face	–	+	+	+	+	–	+	+	+	+	+
Prominent mandibular angle	–	–	–	+	+	–	+	–	–	–	–
Oval face and prominent chin	+	+	+	+	+	+	–	+	+	+	–
Microcephaly	+	+	+	+	+	+	–	–	+	+	–
Hypertelorism	+	+	+	+	+	–	+	–	+	+	+
Ears posteriorly rotated/ conchae anomaly	+	+	+	–	–	+	+	+	+	+	+
Asymmetric smile	n/a	n/a	n/a	n/a	n/a	n/a	n/a	n/a	–	–	–
*Neurodevelopment*											
Hypotonia (mild to moderate)	–	–	–	+	+	–	+	–	+	+	–
Oropharyngeal dysphagia	+	+	–	–	–	+	–	–	–	–	–
Moderate high-frequency neurosensory hearing loss	+	+	+	–	–	+	–	–	–	–	–
*Developmental delay*											
Cognitive impairment	+	+	+	+	–	+	+	+	+	+	+
Impairment of speech expression and perception, articlulation difficulties, disordered intonation, prosody	+	+	+	+	–	+	+	+	+	+	+
Behavioral difficulties (attention deficit, withdrawal, hyperactivity, anxiety, ADHD)	+	–	–	–	–	–	+	+	+	+	+
Short stature	–	–	+	+	–	–	–	–	–	–	–
Hypoglycemia	–	–	–	+	–	–	–	–	–	–	–
*Musculoskeletal features*											
Long fingers and toes	–	–	–	–	–	–	5th finger clino-dactyly	–	–	–	–

*ADHD* attention-deficit/hyperactivity disorder

The average age of diagnosis among children in the cited reports was 5.8 years in families from Romania [3], 3.3 years in Russian family, while the only case from Sri Lanka was diagnosed at the age of 4 years [13], and 2.5 years in the Italian case report [14]. Thus, in all described clinical PTLS cases the diagnosis was established in preschool age. The reason for additional examination of children in families with more than one child was the presence of existing children in the family with similar phenotypic characteristics.

Analysis of the phenotypic characteristics of 11 people presented in the discussion revealed micrognathia in 6 patients (54.5%), sloping palpebral fissures in 8 patients (72.7%), wide forehead in 9 patients (81.8%), pointed/ long tip of the nose in 10 patients—90.9%, triangular face in 9 patients (81.8%). Also, in our case “elfin” ears were found in the mother and son. Such abnormal external ear was also described in a family case from Romania in 4 children and in the Italian case [14].

Short stature was reported only in two children from a family case from Romania. Weight deficiency among the children presented for discussion was observed in 4 children (44.4% among children). Weight loss was not observed among adult patients. Some (45.4%) of the children described and compared in this article had hypotension of varying severity. There was a delay in psychoverbal development and neuropsychic development in 10 people, which amounted to 90.9% of cases.

Thus, the discussed PTLS patients in most cases have characteristic facial features (sloping palpebral fissures, wide forehead, triangular face, pointed nose tip) as well as disturbances in psychoverbal development.

Presented here clinical observation of PTLS within one family has the hereditary nature of the pathology not in doubt. The results of the molecular genetic examination made it possible to differentiate it from other rare conditions. It should be noted that the differential diagnosis of PTLS is extensive since all the presented clinical manifestations can be observed individually or in combination in individuals with other genomic disorders, such as Smith-Magenis syndrome (SMS) (microdeletion of the copy number variant in the 17p11.2 region), Williams-Beuren syndrome, brachydactyly syndrome—intellectual deficit (del 2q37), Prader-Willi syndrome or Sotos syndrome. The first disease with which it is necessary to carry out a differential diagnosis is SMS, a rare syndrome with similar clinical manifestations and involvement of chromosome 17 (deletion or point mutation involving the *RAI1* gene) [15]. Both PTLS and SMS associated copy number variation can arise from a non-allelic homologous recombination event involving the 17p11.2 region, in which the *RAI1* gene is found. Other genes that have been identified in this region include *SREBF1*, *DRG2*, *LLGL1*, *SHMT1*, and *ZFP179* [11, 15, 16]. In the differential diagnosis of these syndromes the most significant are laboratory molecular genetic research methods CMA and MLPA. They are not routine in the practice of a pediatrician working in Russian provinces and are used in the laboratories of federal medical genetic centers and are prescribed by a geneticist only after concise medical genetic counseling of the family. The latter is carried out by the recommendation of the attending physician (pediatrician, neurologist) who suspects the hereditary nature of the disease. In the presented family the diagnosis of PTLS was made only after the birth of the third (healthy) child to this family. The lack of specificity of the clinical picture in PTLS indicates the need for a more careful assessment of the clinical phenotype of the child and other family members during examination. Medical genetic counseling must be carried out in patients with developmental delay, neurobehavioral differences or neurological symptoms, combined with minor developmental anomalies.

It should be noted that this syndrome occurs sporadically (de novo) and can be inherited in an autosomal dominant manner. If 17p11.2 duplication is found in a proband and is not identified in either parent, the risk of future pregnancies may be slightly higher than in the general population (though still < 1%) due to the possibility of somatic and/or fetal mosaicism of the parents [12]. If one of the parents in the family has this 17p11.2 microduplication, the risk of inheritance for each siblings is 50%, which is typical for the described clinical cases. It is not possible to reliably predict the exact clinical phenotype of individuals who inherit the PTLS associated duplication. In the described cases the mother of the children did not manifest all clinical features while in her children detailed clinical findings were observed. In the case of timely diagnosis in the family prenatal testing or preimplantation genetic testing using CMA to detect 17q11.2 duplication is conceivable.

Despite the speech and cognitive impairments, carrying out of timely rehabilitation using a multidisciplinary approach result in a positive developmental dynamic of such children, as well as favorable adaptation and societal integration, which is also confirmed in our case.

## Conclusions

Children with Potocki-Lupski syndrome have a specific clinical phenotype: craniofacial dysmorphism, motor and neurological manifestations with significant clinical polymorphism. The presented clinical cases confirm that pediatricians and other specialists need to pay attention to the phenotype of the child and family members (small developmental anomalies), features of the neurobehavioral and neuropsychiatric development of the child, which may alert the possible genetic causes of the disease. This will allow earlier diagnosing of the condition. Early diagnosis and a multidisciplinary approach in such patients allow one to potentially implement a significant expansion of the child's ability to adapt and socialize and start early rehabilitation, which ultimately is the key to a high quality of life for the patient and his parents.

The diagnosis in the family enables timely prospective medical genetic counseling, helps the family to make an informed decision regarding reproductive behavior, and to plan pre-implantation and prenatal diagnostics in a timely manner.

## Data Availability

Not applicable.
